# Toward a phenological mismatch in estuarine pelagic food web?

**DOI:** 10.1371/journal.pone.0173752

**Published:** 2017-03-29

**Authors:** Xavier Chevillot, Hilaire Drouineau, Patrick Lambert, Laure Carassou, Benoit Sautour, Jérémy Lobry

**Affiliations:** 1 Irstea, UR EABX, Cestas, FRANCE; 2 Université de Bordeaux, UMR CNRS 5805 EPOC–OASU, Station Marine d'Arcachon, Arcachon, France; Technical University of Denmark, DENMARK

## Abstract

Alterations of species phenology in response to climate change are now unquestionable. Until now, most studies have reported precocious occurrence of life cycle events as a major phenological response. Desynchronizations of biotic interactions, in particular predator-prey relationships, are however assumed to strongly impact ecosystems’ functioning, as formalized by the Match-Mismatch Hypothesis (MMH). Temporal synchronicity between juvenile fish and zooplankton in estuaries is therefore of essential interest since estuaries are major nursery grounds for many commercial fish species. The Gironde estuary (SW France) has suffered significant alterations over the last three decades, including two Abrupt Ecosystem Shifts (AES), and three contrasted intershift periods. The main objective of this study was to depict modifications in fish and zooplankton phenology among inter-shift periods and discuss the potential effects of the resulting mismatches at a community scale. A flexible Bayesian method was used to estimate and compare yearly patterns of species abundance in the estuary among the three pre-defined periods. Results highlighted (1) an earlier peak of zooplankton production and entrance of fish species in the estuary and (2) a decrease in residence time of both groups in the estuary. Such species-specific phenological changes led to changes in temporal overlap between juvenile fish and their zooplanktonic prey. This situation questions the efficiency and potentially the viability of nursery function of the Gironde estuary, with potential implications for coastal marine fisheries of the Bay of Biscay.

## Introduction

Over the last few decades, many authors have reported ongoing biological effects of climate change [1–6] impacting species and their habitats and shaping the structure of ecological communities as well as overall functioning of ecosystems [1,7–10]

Among those effects, alterations of species phenology are assumed to have major consequences on ecosystems [3,11]. Phenology is defined as the timing of recurring (seasonal) animal and vegetal life cycle processes [6], including spawning, migrations, bud break, growth and flowering, in response to seasonal variations in their environment [see 12 for a review]. The synchronization between biological processes and seasonal environmental fluctuations has crucial importance for individuals’ fitness, because most species generally rely on limited suitable environmental windows [13]. Most of these phenological events at the species scale are however particularly sensitive to climate change [3,14–16]. In particular, most aquatic animals are ectothermic and their biology and ecology are therefore driven by water temperature. An increase in temperature thus accelerates their metabolism. Consequently, until now, most studies have reported precocious occurrence of life cycle events as a response to climate change and temperature rise [6,17–19], either because of earlier favorable environmental conditions [see exemple of producers in 20,21] or because of phenological modifications in preys or predators dynamics [22]. The timing or, more specifically, the mismatch of species’ life cycle events with favorable environmental conditions, can have various implications at the community, assemblage, and species scales. The interactions between predators and prey at different levels within a food web indeed depend on spatial overlap and temporal synchrony. An increase in time-lag between predator and prey dynamics (occurrence and/or abundance) can lead to so-called mismatches described in the framework of the Match-mis Match Hypothesis (MMH) [23].

Although the Match-mismatch hypothesis is prevalent in the literature, examples actually illustrating it in aquatic ecosystems remain scarce Many reports can be found from terrestrial species and food chains, like in the famous example of oak (*Quercus robur*), moth (*Opheroptera brumata*) and great tit (*Parus major*) [19]. In aquatic environments, Philippart [24] described a mismatch between phytoplankton, bivalve (*Macoma balthica*) and shrimp (*Crangon crangon*) predation induced by mild winter’s temperature in northwestern European estuaries. The earlier spawning period of *Macoma balthica* due to warmer temperature, caused a mismatch with the phytoplankton bloom (not related to temperature) which was then followed by a decrease of *Macoma* food availability and consequentincreased mortality. Furthermore, precocious spawning *Macoma* resulted in an increase of the predation pressure exerted by *Crangon crangon*. In this example, the cold-water species *Macoma* clearly highlights the vulnerability of some species to phenological changes. [19] Coastal environments, especially temperate ones, are considered among the most vulnerable to climate change pressure and mismatch risk [17]. It is therefore essential to examine potential phenological variations in these systems, as such variations can have substantial consequences on trophic relationships and coastal ecosystem functioning.

This question is even more pressing in estuarine ecosystems as they represent important nursery grounds for many juvenile fish. The question of potential climate change-induced match-mismatch between fish (predators) and plankton production (preys) in estuaries is therefore crucial. Recruitment at higher trophic levels is indeed sensitive both to the degree of synchronization with pulsed zooplanktonic production [25,26] as well as to the number of recruits becoming mature and joining fishing stocks. Furthermore, for most species, recruitment is also partly depends on an estuarine growth phase [27–30]. Ottersen [31], Durant [32,33] and Beaugrand [34] explored the impacts of phenological mismatch on fish-plankton interactions in coastal areas. Other authors focused on phenological modifications in estuaries using a monospecific approach, for example for the larval herring (*Clupea harengus*) in the St Lawrence estuary [35] or the striped bass (*Morone saxatilis*) in Chesapeake Bay [36]. David [37] and Selleslagh [38], described the seasonal life-cycle of the main zooplankton and fish species, respectively, in the Gironde estuary. To our knowledge, no study has yet combined phenological species evolution with an analysis of its implications in an estuarine ecosystem context.

The Gironde estuary is one of the largest macro-tidal estuaries in Western Europe [39], as well as being one of the main nursery area for juvenile marine fish from the Bay of Biscay [29]. It is also an important migratory corridor for various diadromous fish species (e.g., *Alosa alosa*, *Anguilla anguilla*, *Alosa fallax*, *Salmo salar*, *Acipenser sturio*, *Petromyzon marinus*, *Lampetra fluviatilis*,…[39]). Significant alterations in the Gironde estuary ecosystem structure and functioning have been documented and related to global changes [37,40–43]. David [37] and Chaalali [44] put forward deep changes in the structure, abundance and dynamics of zooplankton and suprabenthic assemblages. Pasquaud [45] also highlighted significant changes in the structure and dynamics of assemblages of small pelagic fish (anchovy, sprat, and shad) in the middle part of the estuary. Chaalali [46], implementing a multivariate analysis of time series (Principal Component Analysis), described two abrupt ecosystem shifts between 1979 and 2009 (circa 1987 and 2001), in relation with hydro-climatic parameters. Chevillot [10], who focused on estuarine fishes, implemented both a multivariate and STARS (Sequential algorithm for testing climate regime shift) [47] approach on a 1985–2014 time series, which allowed to specify shifts times, circa 1988 and 2002, and to define 3 inter-shift periods with significant differences in ecological dynamics and functioning.

Zooplankton (copepods) and suprabenthic (mysids) species [37]) are key species in the Gironde estuarine food web [48]. In this context, moving further from Chevillot et al. [10], the present article aims at depicting phenological modifications between the aforementioned intershift periods for fish and zooplankton species and at assessing the potential resulting mismatches at a community scale. We focused on the 1985-2010time series. Phenological evolutions of considered species were analyzed by tracking changes in yearly patterns of abundance in long-term scientific survey data. These patterns were estimated using a Bayesian hierarchical model with hidden Markov process. Traditional phenological indicators were computed in this Bayesian context to characterize these changes. This allowed to us to describe changes in the time overlap of predator-prey couples in the estuarine area, leading to discuss implications of those changes and potential mismatch between prey and predators for the Gironde estuary food web dynamics.

## Materials and methods

### Study area

**Fig 1:**
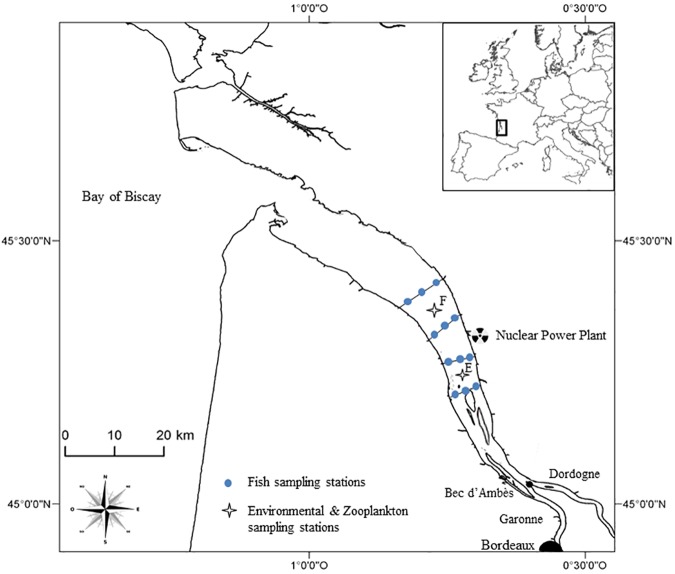
Study area in the Gironde estuary, southwest France.

The Gironde estuary (45° 20’N, 0°45’W, central point) is 70 km long from the mouth (Royan) to the confluence of the Garonne and Dordogne Rivers (Bec d’Ambès) (Fig 1). This macro-tidal estuary is highly turbid. Concentrations of suspended particulate matter (SPM) often exceed 500 mg l^-1^ [49,50]. Mean freshwater discharge approximates 953m^3^ s^-1^ (1960 to 2005; [51]). Main biological compartments (especially fish and zooplankton) and environmental conditions characterizing the Gironde estuary are regularly sampled since 1979 by Irstea, especially in the poly-mesohaline zone. Samples retained for this study originated from the middle reaches of the estuary (Fig 1).

### Hydro-climatic data

#### Data sources

Daily estuary water discharge data were provided by the Bordeaux Port Authority (Bordeaux Port Atlantique). Air temperature data were provided by the Méteo France Center of Mérignac (data collected at the Pauillac meteorological station; middle estuary). These daily air temperature records were measured locally from 1985 to 2010, and are assumed herein to combine effects of global patterns and and local variations.

Salinity data were obtained from several routine monitoring programs of the Gironde estuary, including the Blayais nuclear power plant ecological survey (Irstea-EDF) and the SOMLIT monitoring program (Service d’Observation en Milieu Littoral, INSU-CNRS, http://somlit.epoc.u-bordeaux1.fr, see in [52,53]). In the later program, water samples were collected monthly from March to November (at 1 m below the water surface and 1 m above the bottom, at 3 h intervals during the tidal cycle) in two sampling stations (E and F; Fig 1). These three hydro-climatic parameters (estuary water discharge, air temperature and salinity) are considered important environmental cues triggering yearly phenological patterns of species [37,38,45,54].

#### Recent hydro climatic patterns

**Fig 2:**
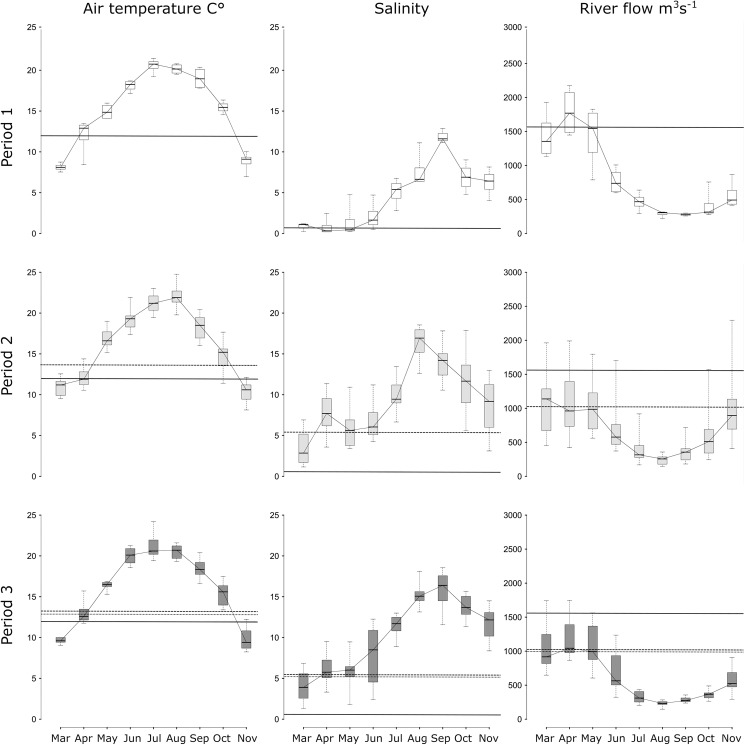
Seasonal changes in abiotic environmental variables in the Gironde estuary between 1985 and 2010.

Over the [1985–2010] period covered by our study, Chaalali et al. [46] previously reported significant long term trends for the three aforementioned hydro-climatic variables. They specifically observed a significant increase in air temperature and estuarine water salinity, which the authors associated to a decrease of water discharge. Meanwhile, these variables exhibited seasonal modifications (Fig 2), withthe observed long term trend [45] especially related to conditions observed in spring, i.e., from March to May. Mean spring values of air temperature and salinity indeed increased after 1988 while mean spring river flow decreased.

The three columns correspond to the mean seasonal pattern of the air temperature, salinity and river flow, respectively, for each of the three periods considered in our study (period 1 1985–1988 top row: white boxplot, period 2 1989–2002 middle row: light-grey boxplot, and period 3 2003–2010 lower row: dark-grey boxplot). The thick line of the boxplot presents the median, the box to the first and third quartile and the whiskers to the 0.025 and 0.975 percentile. Horizontal dotted-line shows the mean value of the “spring months” (March, April and May) for the period considered.

#### Fish data

Fish data were obtained from the ‘Transect’ surveys implemented by the French National Research Institute of Science and Technology for Environment and Agriculture (Irstea) since 1979 to monitor small fish species or juvenile stages of larger species and shrimps around the Blayais nuclear power plant [38,55]. Fish surveys were carried out on a monthly basis along 4 transects, each consisting in three sites distributed along a transversal axis from one bank to the other (Fig 1). At each site, two samples were collected simultaneously near the surface and near the bottom [38,55]. Trawls lasted five to seven minutes and were all performed during daytime, between the halfway stage of the flood tide and the high tide slack, with gear being towed against the current (see for instance [38,55] for details of the protocol).

#### Zooplankton data

Zooplankton data were obtained from the same Blayais ecological nuclear power plant ecological survey that provided fish data (see details in [37,44,46]). Samples were collected monthly from March to November since 1979 (at 1 m below the water surface and 1 m above the bottom, and at 3 h intervals during the tidal cycle). Two sampling stations (E and F; Fig 1) were selected as part of our study area.

#### Data pretreatment

Data for estuary water discharge, temperature and salinity were monthly averaged from 1985 to 2010. Fish abundance from all sampling stations and depths were averaged to obtain one mean value per month and per year. Only the most frequent fish species were considered: only species whose frequency of occurrence (measured as the proportion of samples in which the species was actually caught) exceeded 2% were retained (Table 1). We focused on the period 1985–2010, as before 1985, no analysis was possible due to too many missing data.

Studying phenology implies to work at a temporal scale consistent with ecological events. Therefore, we chose to work with ‘ecological’ years rather than calendar years. Consequently, for the species which are present with maximal abundance in the estuary during spring and summer (i.e., all species except Liza ramada and Anguilla Anguilla), ecoligocial year x corresponds to the months from March of year x to February of year x+1, while for species present with maximal abundance in winter (i.e., Liza ramada and Anguilla Anguilla), ecological year x corresponds to months from July of the year x to June of year x+1.

**Table 1:** List of the fish species selected for the present study. The term ‘frequency” corresponds to the frequency of occurrence in the sampling data [39] between 1985 and 2010.

Species	Common name	Frequency(%)
Alosa alosa	Allis shad	9.5
Alosa fallax	Twaite shad	27.0
Anguilla anguilla	Eel	25.3
Argyrosomus regius	Meagre	3.0
Dicentrarchus labrax	Seabass	27.5
Engraulis encrasicolus	Anchovy	34.8
Gasterosteus aculeatus	Stikleback	21.2
Liza ramada	Mullet	45.5
Osmerus eperlanus	European smelt	13.44
Platichthys flesus	Flounder	4.4
Pomatoschistus sp	Goby	73.5
Solea sp	Sole	3.3
Sprattus sprattus	Sprat	19.9
Syngnathus rostellatus	Pipefish	39.8

Zooplankton data were monthly averaged for the same study period (1985–2010) to consider tidal and vertical variability (data after 2010 were not yet available for analysis). We focused on the five dominant species (regarding their abundance and fodder characteristic [46]) of this zooplankton compartment: *Eurytemora affinis*, *Acartia bifilosa*, *Acartia tonsa*, *Neomysis integer* and *Mesopodopsis slabberi*.

### Data analysis

**Fig 3:**
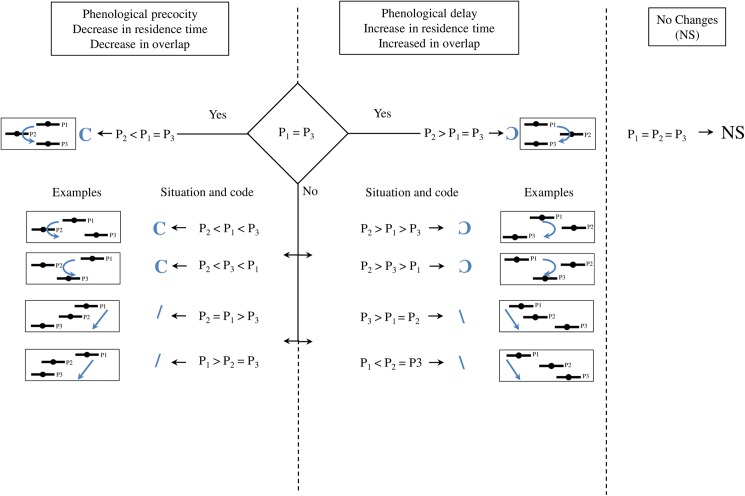
Decision tree

**Fig 4:**
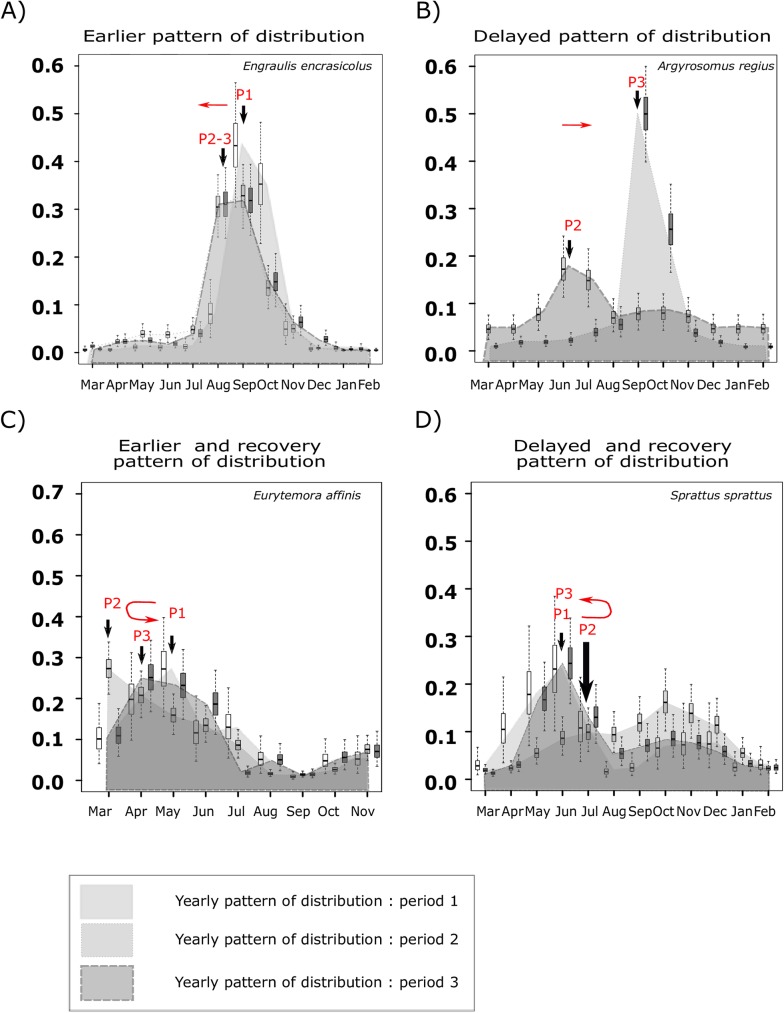
Example of four modifications of yearly distribution patterns among periods: the four situations illustrate the different types of phenological changes accounted for in this study. The red arrows correspond to peak of abundance of species for the period 1 to 3 (noted: P1, P2, P3). A) The annual peak of E. encrasicolus abundance shiftedf earlier in the year for the period two and three. B) The annual peak of A. regius abundance shifted later in the year for the period three. C) The annual peak of E. affinis shifted earlier in the year for the period two and returned near the first situation during the third period. D) The annual peak of S. sprattus shifted later in the year for the period two and returned near the first situation at the third period (note: for this species, only the first peak of presence in the estuary was considered).

Phenological modifications were explored by estimating yearly patterns of abundance for each species and period. Those patterns were estimated using a flexible Bayesian model. Such a model was used because it facilitates uncertainty quantification and therefore the comparison among periods. Moreover, our model allows flexibility for the use of non-normally distributed data, as compared to normal distributions assumptions usually associated with other modeling techniques used in literature to model phenological event. Two questions were addressed using this approach: (i) did phenological modifications occur for each species individually and, if so, which kind of modifications (Early arrival? late departure? longer residence time?), (ii) what were the possible consequences of those modifications on trophic interactions, through the analysis of temporal overlap of estuarine residency between prey and predators? For both questions, we classified phenological and trophic situations into four trajectories (Figs 3 and 4):

- The species peak of abundance was significantly earlier in period 2 than in periods 1 or 3 (symbol C)
- The species peak of abundance was significantly later in period 2 than in periods 1 or 3 (symbol Ɔ)
- The species peak of abundance was significantly earlier in period 1 than in the two other periods (symbol \)
- The species peak of abundance was significantly later in period 1 than in the two other periods (symbol /)

Similar trajectory scenarios were used to address overlap between predators and prey.

### Bayesian hierarchical models with hidden Markov process

We denote A_s_(y,m) the abundance observed for species s in year y and month m. The total abundance $A_{s}(y)=\sum_{m=1}^{12}A_{s}(y,m)$ corresponds to the total abundance observed in year y. A yearly pattern (S1A Fig) P_s_(y) is defined as:

$$P_{s}(y)=\left\{\frac{A_{s}(y,1)}{A_{s}(y)},\ldots ,\frac{A_{s}(y,12)}{A_{s}(y)}\right\}$$

i.e. the vector of proportions of the total abundance of species s that is observed each month of year y. By construction, the elements of P_s_(y) sum to one. If year y belongs to an inter-shift period j (defined as a set of successive years between ecological shifts: 1985–1988, 1989–2002, 2003–2010), P_s_(y) is assumed to follow a Dirichlet distribution around the average yearly pattern of j:

$$P_{s}(y)∼Dirichlet(\lambda_{s}\cdot \{a_{s,j,1},\ldots ,a_{s,j,12}\})$$

where a_s,j,m_ is the species s average proportion in month m during period j. *λ*_*s*_ is often called Dirichlet concentration parameter (a value below 1 leads to sparse distributions while a value above 1 leads to dense distributions, i.e. closed to the average proportions).

For a given species s, a change between two average patterns from period j_1_ and j_2_ is assumed to be significant when there is at least one month m for which a_s,j1,m_ and a_s,j2,m_ are significantly different (i.e. 95% credibility intervals do not overlap).

The model (S2 Fig) aims to estimate those average yearly patterns {*a*_*s,j*,1_,…,*a*_*s,j*,12_} of distribution of abundance for each species s (fish and zooplankton) at each defined period j to detect significant modificationsamong periods.

We used flat priors for *λ*_*s*_ and {*a*_*s,j*,1_,…,*a*_*s,j*,12_}:

$$\lambda_{s}∼Unif(0.3,2000)$$

$$\left\{a_{s,j,1},\ldots ,a_{s,j,12}\}∼Dirichlet(\left\{1/12,\ldots ,1/12\right\}\right)$$

### Bayesian inference

The S2 Fig presents the Jags code. All computations were performed within R software [56]. Bayesian posterior distributions were approximated via Monte Carlo–Markov chain methods through the open-source Jags software [57]. R and jags interface was conducted using the library runjags [58]. Three MCMC-independent chains were used. For each chain, the first 10,000 iterations were discarded. After this “burn-in” period, inferences were derived from a sample of 3 × 50,000 iterations. All the modeling results were evaluated by the Gelman-Rubin diagnostic ([59], R ratio <1.05 for all variables). The parameters posterior distributions were used to derive their 95% credibility intervals which were then compared to depict significant differences among the three periods.

#### Species-specific phenological indicators

Five indicators usual in the literature about phenology were calculated at each iteration of the model (see review in [16,60]). We first computed the central tendency (T_s,j_) of each species s distribution during period j. It corresponds to the timing of the monthly peak of abundance of the species in the Gironde estuary (i.e. the month for which the estuarine abundance of species s is highest during the year). It was computed using the month co-ordinate of the center of gravity of the area below graphs of yearly pattern:

$$T_{s,j}=\frac{\sum_{m=1}^{12}m\cdot a_{s,j,m}}{\sum_{i=1}^{12}a_{s,j,m}}$$ (1)

This index is sensitive to changes in the timing of the seasonal cycle [17,61]. However, many coastal temperate ecosystems can exhibit two production peaks: one in spring and the other in autumn. The average yearly pattern over the whole dataset period (1985–2010) for each species was used to determine whether species were unimodal (with only on peak of abundance in the year) or bimodal (exhibiting two peaks of abundance during the year, generally in spring and summer/autumn). In the cases of unimodal species, the timing of the seasonal peak was calculated throughout the entire ‘ecological’ year, whereas for bimodal species, the timing of the seasonal peak was calculated separately for the first six months and the last six months of the year.

For each species s, we also calculated Quantile 10_s,j_ (respectively Quantile 90_s,j_) which corresponds to the date when 10% (respectively 90%) of total annual abundance is reached in period j according to the average pattern. Quantile 10_s,j_ are calculated in months and we used a linear approximation between a_s,j,m_ and a_s,j,m+1_. Quantiles 10 s,j were defined in this study as respectively the “beginning” and the “end” of species occurrence period in the estuary. Similarly, Quantile 50_s,j_ correspond to middle time of the occurrence period. Quantile 50_s,j_ and T_s,j_ are rather similar, however they can provide distinct signals when yearly distribution is asymmetric. Finally, using the average patterns, we also calculated the residence time (Tr_s,j_) as the minimum number of months required to observe 90% of the occurrence of species s in the estuary during the year.

For each indicator, a difference between two periods was assumed to be significant if the corresponding 95% credibility intervals did not overlap (S3 Fig, synthesis in Table 2).

**Table 2:** Synthesis of phenological indicators results: symbols “C”, “Ɔ”, “/”, “\” and “NS”refer to decision tree Fig 2. The numbers between brackets correspond to the count of significant pairwise differences among periods for each couple species/indicators (T: central tendency, Quantiles 10, 50 and 90, Tr: time of residence). Light-grey boxes with “NS” show the situations with no phenological changes detected among periods.

species\ Indicators		T	*Quantile* 10	*Quantile* 50	*Quantile* 90	Tr
*Fish*	*L*. *ramada*	NS	NS	NS	NS	NS
	*A*. *anguilla*	NS	NS	NS	NS	NS
	*A*. *alosa*	/ _(3)_	/ _(2)_	/ _(3)_	/ _(2)_	Ɔ _(1)_
	*A*. *fallax*	/ _(2)_	NS	/ _(2)_	/ _(2)_	/ _(2)_
	*E*. *encrasicolus*	/ _(2)_	/ _(2)_	/ _(2)_	NS	\ _(2)_
	*O*. *eperlanus*	NS	NS	NS	NS	NS
	*G*. *aculatus*	/ _(2)_	NS	/ _(1)_	/ _(2)_	/ _(2)_
	*P*. *flesus*	NS	NS	NS	NS	NS
	*Pomatoschistus sp*.	NS	NS	NS	Ɔ _(1)_	Ɔ _(1)_
	*A*. *reguis*	\ _(1)_	\ _(1)_	\ _(1)_	/ _(1)_	/ _(1)_
	*Dicentrarchus sp*.	C _(1)_	NS	C _(1)_	NS	NS
	*Solea sp*.	NS	NS	NS	NS	NS
	*S*. *sprattus*	Ɔ _(1)_	Ɔ _(1)_	Ɔ _(1)_	NS	NS
	*Syngnathus sp*.	C _(1)_	C _(1)_	C _(1)_	C _(1)_	C _(1)_
*Zooplankton*	*E*. *affinis*	C _(1)_	C _(1)_	NS	NS	NS
	*A*. *bifilosa*	/ _(2)_	NS	/ _(1)_	NS	NS
	*A*. *tonsa*	NS	NS	C _(1)_	C _(1)_	NS
	*N*.*integer*	/ _(1)_	NS	/ _(1)_	NS	NS
	*M*. *slabberi*	NS	/ _(1)_	NS	NS	NS

#### Multispecific phenological indicators

Literature documented 46 situations of predator/prey interactions [45,48,62] between fish and zooplankton in the Gironde estuary. An interaction is defined as a co-occurrence [32] of prey and predators for each period. The percentage of overlap (S4 Fig, synthesis in Table 3) O_j,s1,s2_ between distributions of prey s1 and predator s2 during period j is defined as:

$$O_{j,s1,s2}=\sum_{m=1}^{12}min(a_{s1,j,m},a_{s2,j,m})$$

We assumed that this indicator is a proxy of the potential trophic interaction magnitude between species s1 and s2.

**Table 3:** Synthesis of predator/prey interaction results: symbols refer to decision tree in Fig 2. Dark-grey boxes show the undocumented situations of predatory/prey interactions?. Light-grey boxes show the situations without changes in predator/prey overlap.

Fish sp.\ zooplankton Sp.	*E*. *affinis*	*A*. *bifilosa*	*A*. *tonsa*	*N*.*integer*	*M*. *slabberi*
*L*. *ramada*	NS	NS			
*A*. *anguilla*				NS	NS
*A*. *alosa*	NS	NS	NS	**Ɔ**	NS
*A*. *fallax*	NS	NS	**Ɔ**	NS	NS
*E*. *encrasicolus*	NS	**Ɔ**	NS	**Ɔ**	NS
*O*. *eperlanus*	NS	**Ɔ**	NS		
*G*. *aculatus*				NS	NS
*P*. *flesus*				NS	NS
*Pomatoschistus sp*.	NS	NS	NS	**/**	NS
*A*. *reguis*	**/**	**/**	**\**	**/**	**/**
*Dicentrarchus sp*.	NS	NS	NS	**Ɔ**	NS
*Solea sp*.					
*S*. *sprattus*	**C**	NS	**Ɔ**		
*Syngnathus sp*.				**/**	**Ɔ**

### Formulation of intelligible comparisons

The combinations of 19 species with five phenological indicators led to 95 situations of potential species-specific phenological changes detection. Moreover, 46 situations of predator/prey interactions are documented. Accordingly, it was necessary to extract from those multiple situations an intelligible comparison of phenological and trophic situations among periods. We chose to summarize the information into the four previously mentioned trajectory scenarios of phenological evolution. The four trajectory were coded as “C”, “Ɔ”, “/”, and “\” and presented in a decision tree (Fig 3 examples from data Fig 4).

For the phenological indicators T and quantiles 10, 50 and 90, the “/” corresponds to phenological precocity across periods. For the residence time and overlap, the “/” corresponds to an increase of both parameters across periods.

For the phenological indicators T and quantiles 10, 50 and 90, the “\” corresponds to phenological delay across periods. For the residence time and overlap, the “\” corresponds to a decrease of both parameters across periods.

For the phenological indicators T and quantiles 10, 50 and 90, the “C” corresponds to phenological precocity of the second period compared to the first and third periods, withhe third period presenting or not a similar situation than the first period. For the residence time and overlap, the “C” corresponds to a decrease in the second period compared to others, and a re-increase in the third period.

For the phenological indicators T and quantiles 10, 50 and 90, the “Ɔ” corresponds to phenological delay of the second period compared to the first and third periods, the third period presenting or not a similar situation than the first period. For the residence time and overlap, the “Ɔ” corresponds to an increase in the second period compared to others, and a re-decrease in the third period.

Situations in which no phenological change was detected were noted with “NS” (No Significant changes).

The different situations of phenological evolution among the three periods P1, P2 and P3 are summarized into four trajectories [nomenclature given in the text]. The decision tree presented each trajectory with its code and an example (referred to the S3 and S4 Figs). Situations are grouped by their ecological meaning: “phenological precocity”, “phenological delay” and “no phenological change”). Note that symbols used refer to the dynamic of blue arrows.

## Results

### Yearly patterns

The yearly patterns for each zooplankton and fish species are presented in S1 Fig. Among the 19 species, 14 showed significant modifications of their yearly pattern (S1 Fig) for at least one indicator. The five phenological indicators and their credibility intervals used to characterize changes in yearly patterns are presented in S3 Fig and summarized in Table 2. To ensure that the observed significant changes did not occur by chance, we compared the number of observed changes with the theoretical number of changes that would have occurred only by chance. For each species/indicator couple, three pairwise differences were theoretically possible (period 1 ≠ period 2 ≠ period 3, except for A.regius which did not occur in the middle part of the estuary during the first period, so that only one difference was possible). A total of 55 pairwise differences (18 species * 3 pairwise comparisons = 54 + 1 species (Meager)* 1 pairwise comparison) were possible for each phenological indicator. The sum of pairwise comparisons counted for each indicators led respectively to 17, 9, 15, 10 and 10 differences (Table 2). This corresponds to 30%, 16%, 27%, 18% and 18%, respectively, of the theoretical possible situations. All five indicators were therefore greater than the random threshold of 5%.

### Monospecific phenological indicators

We tested 95 cases (19 species x 5 phenological indicators) of potential phenological change. Amongst these 95 possible cases, 45.3% exhibited significant changes. Among the 19 species studied, five (L. ramada, A. Anguilla, O. eperlanus, P. flesus, and Solea sp.) exhibited no phenological modification. Regarding the first four indicators (central tendency, Q10, Q50 and Q90), we observed significant modifications of species’ patterns in almost half of the cases (27 upon 56) for fish and zooplankton (9 upon 20). In most cases (20 cases upon 27 for fish and 9 upon 9 for zooplankton), these changes are associated with earlier occurrences in period 3 compared to period 1 and/or 2 (/ and C patterns). These modifications were allocated for 23.15% to precocity symbols (/), 11.57% to earlier peak in period 2 (C), 6.3% to later peak in period 2 (Ɔ), and 4.6% to lag symbols (\). Considering only the “time of residence” indicator (19 cases), seven cases (37%) presented significant modifications, and all concerned fish. The most frequent modification was a decrease in the time of residence (the “/”symbol represented 42.7% of the 7 significant modifications). Among the five phenological indicators used, the indicators of central tendency and quantile 50 were those presenting the highest number of modifications. They were modified for 58% of species. Each of the three other indicators represented 7.4% of the total modifications and they were modified for 35% of species.

### Co-occurrences between predators and prey

Significant changes in patterns of co-occurrences (S4 Fig) are summarized in Table 3. For each situation, a symbol was assigned (Fig 3) to account for changes in the indicators among periods. Over the 46 cases of predator/prey relationships tested, we observed 16 cases (35% of possible interactions) that presented at least one significant change between two periods. In these situations, overlaps were modified from 10% to 45% (Table 4). Eeight of them were associated to an increase followed by a recovery (Ɔ), six others to a decrease (/), one to an increase (\), and one to a decrease followed by a recovery (C). These modifications in overlap were observed when both predator and prey exhibited phenological changes (94% of the cases of trophic modifications).

**Table 4:** Magnitude of overlap modification between predator and prey: Values extracted from S4 Fig indicate the magnitude of overlap changes between predator/prey couples. Dark-grey boxes show the undocumented situations of predatory/prey interactions. Light-grey boxes show the situations without changes in predator/prey overlap.

Fish sp.\ zooplankton Sp.	*E*. *affinis*	*A*. *bifilosa*	*A*. *tonsa*	*N*.*integer*	*M*. *slabberi*
*A*. *alosa*	NS	NS	NS	27% -> 50%	NS
*A*. *fallax*	NS	NS	60% -> 27%	NS	NS
*E*. *encrasicolus*	NS	68% -> 46%	NS	25% -> 45%	NS
*O*. *eperlanus*	NS	60% -> 45%	NS		
*Pomatoschistus sp*.	NS	NS	NS	50% -> 30%	NS
*A*. *reguis*	50% -> 25%	64% -> 25%	30% -> 80%	70% -> 25%	60% -> 27%
*Dicentrarchus sp*.	NS	NS	NS	60% -> 75%	NS
*S*. *sprattus*	65% -> 40%	NS	23% -> 45%		
*Syngnathus sp*.				50% -> 25%	60% -> 50%

## Discussion

It is now unquestionable that global changes can significantly affect species’ phenology [4,11,12,63,64]. Previous studies on phenological changes in temperate coastal and estuarine ecosystems have mostly focused on monospecific changes, and species from low trophic levels, such as primary producers [65] or zooplankton [66]. Attrill and Power [67] and Sims [68] however studied climate change impacts on estuarine nursery functions in terms of species growth and spawning time, but trophic implications of those impacts were not explicitly addressed.

Relationships between juvenile fish and zooplankton in estuarine food webs are of essential ecological interest, since estuaries are major nursery areas for many marine fishes [27,69,70]. Our study examined the evolution of phenological patterns for juveniles of several fish species and their prey in the changing climatic and hydrological environment of the Gironde estuary. Chaalali [46] and Chevillot [10] highlighted abrupt ecological shifts in the Gironde estuarine ecosystem over the last three decades. The periods between these shifts (inter-shift periods) exhibited significantly different ecological communities in terms of relative abundance, diversity and functioning. We chose to compare the phenological patterns between these periods in order to clarify whether the already documented climate change impacts on the Gironde estuary also have phenological implications.

Such a problematic first needed the implementation of an innovative method to address the data in a relevant way.

### An innovative Bayesian framework

A flexible Bayesian method was used to estimate and compare yearly patterns of occurrence in the estuary among three pre-defined ecologically homogeneous intershift periods. The Bayesian model we proposed has several advantages. Indeed, in phenological studies, authors generally use specific phenological indicators (quantiles, central tendency…[60,71]) (1) directly estimated for each year from counting or (2) fitting a Gaussian distribution [60] and then focusing on mean or standard-deviation parameters. Using our modeling framework, it is possible to work directly on the yearly pattern without specifying any distributional assumptions. Traditional indicators can then be calculated based on estimated patterns with associated credibility intervals to detect significant changes. The computation of those credibility intervals accounts for both the inter-annual variability in the abundance patterns and differences in data availability among periods.

We described the distribution of phenological indicators using a credibility interval of 95%. Significant differences between periods were confirmed when there was no overlap of distributions. This means that we used a significance level of 5% in the comparisons. This could appear restrictive in some cases, particularly when non-significant changes may be explained by the shortness of the time series. However, with this significance level, we still detected a number of significant changes in phenological indicators (not by chance), suggesting meaningful ecological modifications in the yearly patterns of abundance of species in the estuarine area.

While our results were clear and unequivocal, the spatial dimension of species interactions could not be taken into account. As our spatial window of observation was limited to the middle reaches of the estuary, we indeed only had a limited view of the species’ seasonal occurrence pattern in the whole estuary. However, the spatial area on which we focused encompasses the 3 main haline zones (from the polyhaline to the oligohaline zone, depending on the seasons). Considering that these zones are the most important in terms of both fish diversity and abundances in estuaries [42], we assumed that the observations we made at that scale are representative of the overall functioning of the water body.

### Changes in the species phenology and trophic consequences

The majority (i.e., two third) of fish species and all zooplankton species exhibited modification in their yearly abundance pattern in the Gironde estuarine nursery ground for at least one indicator. Most of these species occurred earlier in the estuary during the last ‘intershift’ periods than during the first one. This may be explained through an adaptation of species spawning period in order to optimize the timing of migration towards the coast in optimal environmental conditions, such as reported for the anchovy in the Bay of Biscay [72]. Other studies reported precocity in downstream migration of fish (e.i *Platichthys flesus*) driven by rising temperature [e.g. 71]. For copepods and mysids, our study is the first to demonstrate an early peak of production in the Gironde estuary, although similar phenological changes have previously been observed in other aquatic systems for those groups [16,66,73]. Changes in the timing of annual zooplankton production may be explained by the earlier appearance of “spring” warmer environmental conditions [17,74,75]. A similar situation may therefore pertain in the Gironde estuary. In the Gironde, upstream movement of copepods, associated with changes in their ecological niche [44], could contribute to explain observed changes in their production seasonality [76]. Other authors made similar observations for fish in other estuarine systems. For instance, the combination of warming and increase of river and estuary water discharge is a crucial trigger of upstream movements in estuaries for marine fish such as soles and flounders [68] and for diadromous species such as the Alosa sapidissima in northwestern United [77] State. Such impact of environmental shifts on species phenological cycle have been documented in many other aquatic and terrestrial ecosystems [20,21,71,78,79]. However, understanding if a phenological modification is a direct response to environmental modifications or an indirect response to prey availability remains a challenge. In the Gironde estuary, we observed a synchronous modification of the first three major environmental variables (salinity, river discharge and air temperature) in spring (between March and May). Spring temperatures and salinity increased during the last two periods, whereas river discharge decreased. This suggests an “earlier spring” as proposed by Le Treut [80] in the Gironde estuary? Further comprehensive studies about the causal link between earlier spring conditions and phenological observations highlighted in our study therefore appear essential.

For five fish species, no phenological change among the three periods examined were observed *L*. *ramada* and *A*. *anguilla* recruit in the estuary during the winter season, while *O*. *eperlanus*, *P*. *flesus* and *Solea sp*. enter the estuary in spring. Occurrences of these two groups of species in the estuary are therefore driven by different environmental triggers. *O*. *eperlanus* also disappeared from our samplings from 2006 onwards. The very small abundance of *O*.*eperlanus* observed in the last decades [39,43] and the highly fluctuating abundances of flounders in recent years [10,81] together suggest a strong modification in environmental conditions triggering their migration in the estuary. However, we still cannot explain the case of *Solea solea* which exhibited changes in estuarine recruitement timing in other temperate estuarine ecosystems [68,74,78].

Our results also showed that almost 40% of studied fish species exhibited changes in their residence time in the estuary. Although the situations were contrasted among species, most of them appeared to spend less time in the estuary during recent periods. These results are contradictory with local observations on the anchovy which have recently increased in abundance and residence time in the estuary [10]. In our study, anchovy’s time of residence in the estuary also increased from the first to the last period, unlike most of the other species examined. Considering the whole set of indicators, we can assume that the Gironde estuary was a more suitable habitat for anchovies in the last two periods, explaining its recent dominance in fish assemblages.

All the fish species we studied use estuaries as nursery grounds or feeding areas during young stages (or entire life cycle for goby) and most of them exhibit a specialist feeding strategy [82]. Zooplankton species, especially copepods and mysid are their preferential prey or represent a significant part of their diet. Trophic interactions between fishes and the aforementioned preys require spatial overlap and temporal synchronicity [15,32]. Synchronicity was assessed in this study by estimating the time overlap between preys and predators yearly patterns. Modifications of this overlap (expressed as a % of overlap) induced by temporal shifts in species-specific yearly patterns can be assumed to affect the overall trophic structure of the estuarine community. Our results therefore highlight that monospecific phenological changes affects temporal synchronicity between fish juveniles and preferential preys. These synchronicities were significantly modified for many predator / prey couples and concerned 9 of the 14 fish species included in the present study. This means that for most of the main fish species, a temporal shift in interaction with at least one of their principal preys, occurred in the last three decades. Among all the tested interactions, one third changed over the last three decades, with half of that proportion due to a decrease in temporal overlap. For these species, we hypothesize that these mismatches could cause a drop in prey availability. This mainly concerns *A*. *fallax*, *Pomatoschistus sp*., *A*. *regius*, *Dicentrarchus sp*. *and Syngnathus sp*. *and*, *to a lesser extent*, *A*. *alosa*, *E*. *encrasicolus and S*. *sprattus*.

As a summary, many fish species seem to spend less time in the estuary and that would lead to a temporal mismatch with their prey. This questions the quality of the Gironde estuarine nursery grounds for marine juveniles [83] especially as their growth and survival is a key factor for a successful subsequent recruitment in marine ecosystems [84]. Further data on marine fish growth within the estuary would therefore provide valuable complementary information to assess the issue of the Gironde nursery quality.Although this study highlighted an alarming situation with regards to the potentiality of the Gironde estuarine ursery grounds, further study should be conducted to explicitly explore the possible limitation of zooplankton resources, and its consequences on the productivity of fish species during their estuarine life through data on juvenile fish condition and growth patterns for example. Measure of fish size, weight, condition and growth, potentially using biomarkers techniques such as fatty acids stable isotopes or sclerochronology, should therefore be implemented to confirm our conclusions and hypothesis.

The aspects of potential degradation of the nursery function (mismatch situations and shorter time of residence) highlighted in our study questioned the more quantitative aspects shown by Pasquaud [45] who linked up the actual marinisation process of the estuary and the increase of juvenile marine fish in the median part with an increase of this nursery function. This raises the question of whether or not this function (in quality and in quantity) of the Gironde estuary is ecologically sustainable.

## Conclusion

The present study confirmed that coastal and estuarine ecosystems can be heavily impacted by global change, affecting not only the structure of biological communities but also ecological patterns and phenology. This can lead to shifts in predator/prey relationships able to cause deep modifications in the structure and functioning of the food web. This can affect the ecological functions associated with estuarine areas for both marine and diadromous fish species. In particular, the synchronistic decrease in species’ residence time and fish predation potential could, in the future, threaten the sustainability of the nursery function of the estuary for many species.

Although its situation is very illustrative of this current problem, the particular case of the Gironde estuary is somehow paradoxical. While studies have highlighted a deep modification in biodiversity during the last three decades [10] associated with an increase in marine juveniles in the area [45], the present study highlighted the mistiming of juvenile fish and their preferential prey in the Gironde estuary, associated with a decrease in residence time and potential mismatch in situations of predation. This situation questions the efficiency—and even the viability–of nursery functions of this system for fish, including marine species. As the Gironde estuary, because its size is one of the most important nursery grounds of the Bay of Biscay, this could have implication for coastal fisheries in this area.

## Supporting information

S1 Fig Mean seasonal pattern of distribution between the three periods. White boxplot, light-grey boxplot and dark-grey boxplot correspond respectively to the 1985–1988; 1989–2002 and 2003–2010 periods. The thick line of e boxplot represents the median of 50,000 iterations, the box corresponds to the first and third quartile and the whiskers correspond to the 0.025 and 0.975 percentile.
(TIF)

S2 Fig Bayesian script of the model. Nby: number of years; nbm: number of month; nbp: number of inter-shift period; esp: Mean; lambda: Dirichlet concentration parameter; alpha: flat prior. The code was performed with Jags software.
(TIF)

S3 Fig Evolution of the five phenological indicators between the tree periods (fish and zooplankton species). Intervals around the mean (black points) represent the intervals of credibility at 95%. For the “Time of residence”, numbers correspond to the number of months of residence of species in the estuary. Letter a, b or c illustrates the significant differences between periods. The symbols refer to the decision tree (Fig 3)
(TIF)

S4 Fig Evolution of the percentage of covering between predators and preys. Intervals around the mean (black points) represent the intervals of credibility at 95%. Letter a, b or c illustrates the significant differences between periods. The symbols refer to the decision tree (Fig 3)
(TIF)
