# Supplementary figures and images for: Toward a phenological mismatch in estuarine pelagic food web?
Source: PLoS One. 2017 Mar 29;12(3):e0173752. doi: 10.1371/journal.pone.0173752 (PMC5371289; doi:10.1371/journal.pone.0173752)

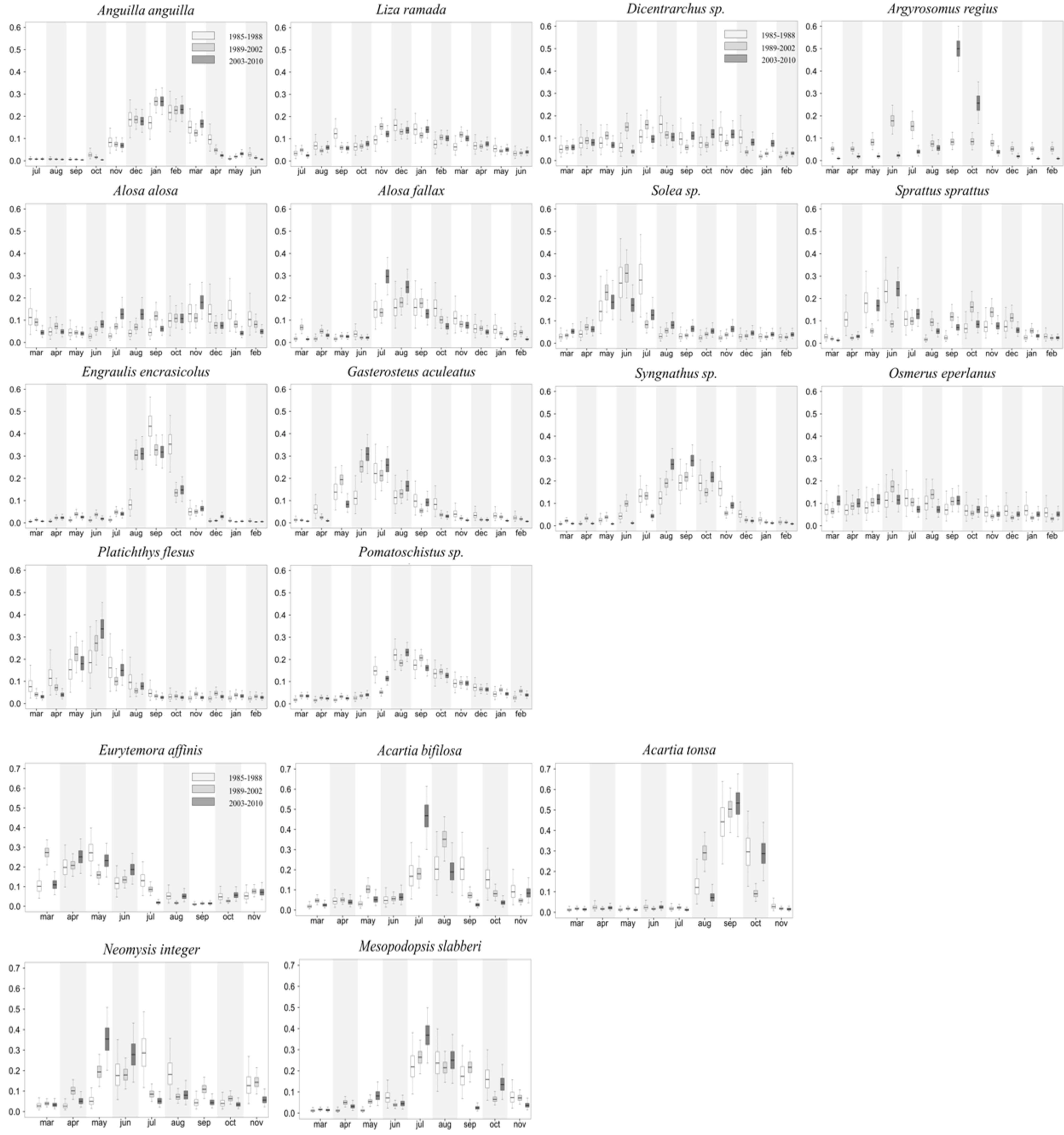

Supplement: S1 Fig — White boxplot, light-grey boxplot and dark-grey boxplot correspond respectively to the 1985–1988; 1989–2002 and 2003–2010 periods. The thick line of e boxplot represents the median of 50,000 iterations, the box corresponds to the first and third quartile and the whiskers correspond to the 0.025 and 0.975 percentile. (TIF) [file pone.0173752.s001.tif]

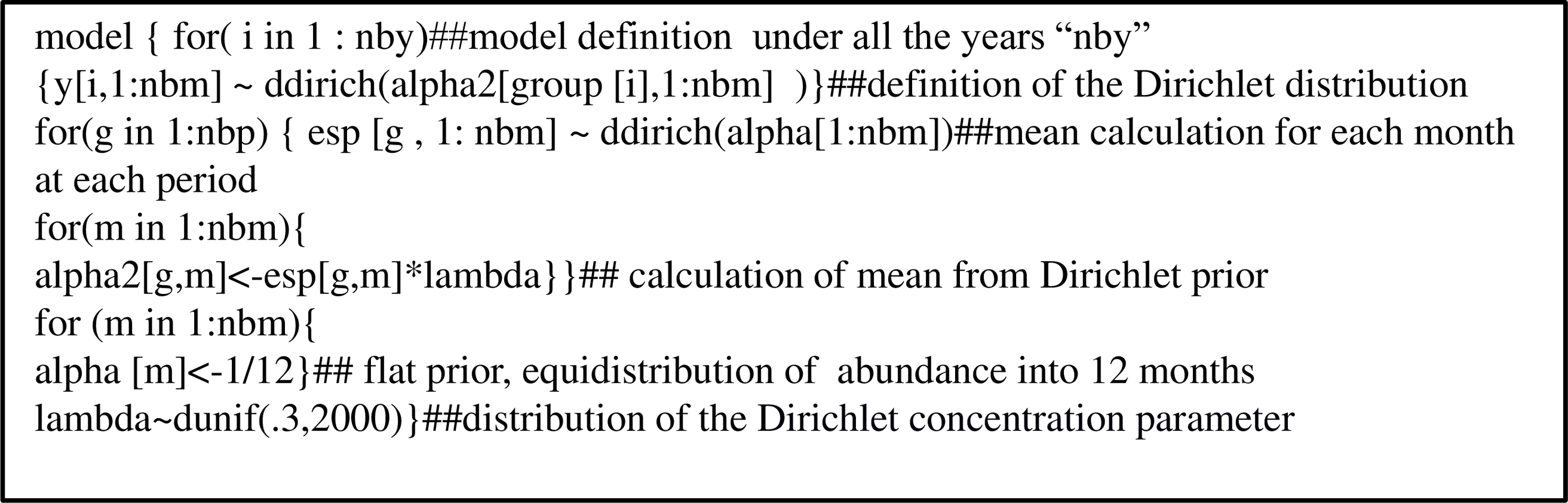

Supplement: S2 Fig — Nby: number of years; nbm: number of month; nbp: number of inter-shift period; esp: Mean; lambda: Dirichlet concentration parameter; alpha: flat prior. The code was performed with Jags software. (TIF) [file pone.0173752.s002.tif]

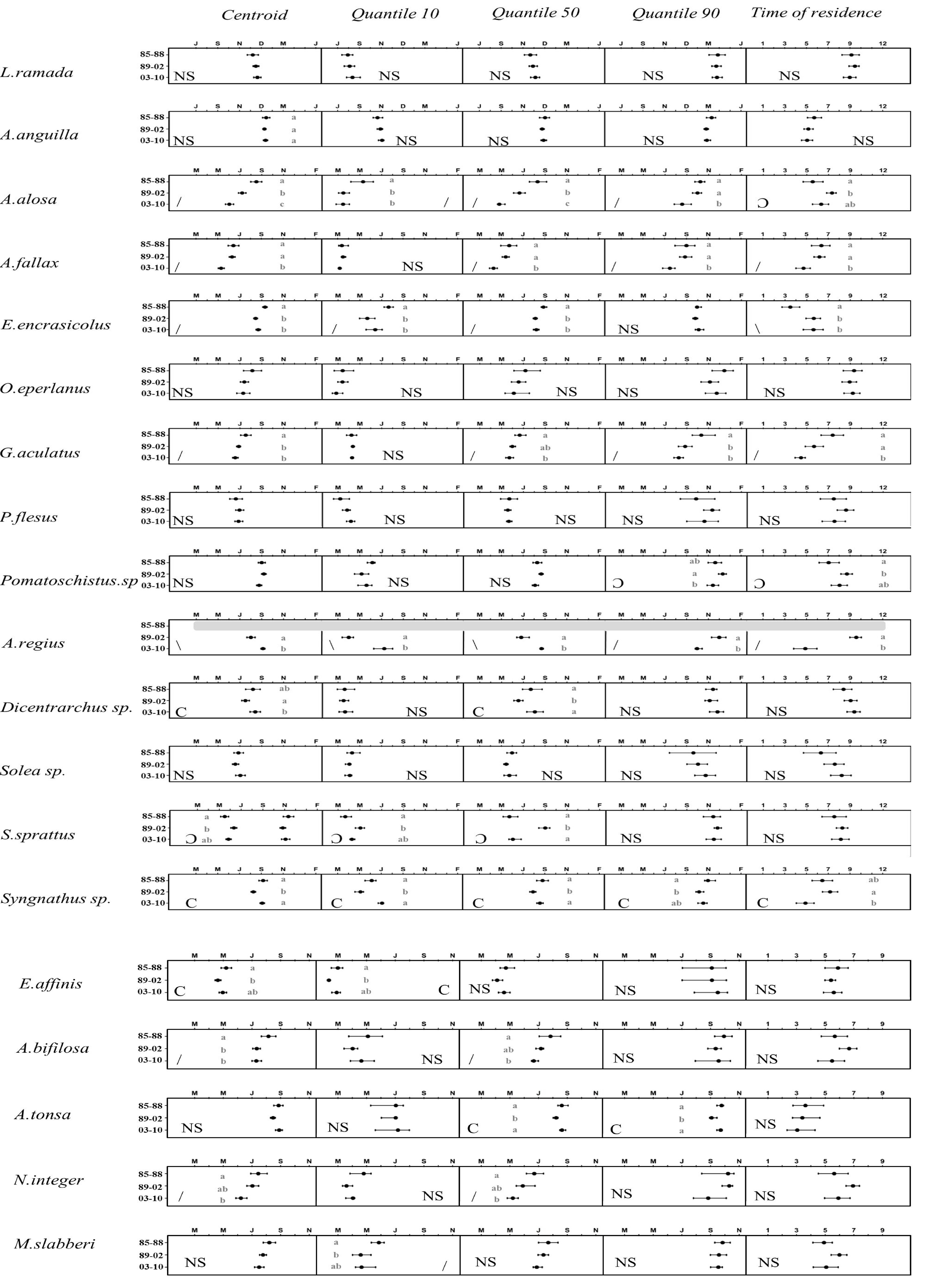

Supplement: S3 Fig — Intervals around the mean (black points) represent the intervals of credibility at 95%. For the “Time of residence”, numbers correspond to the number of months of residence of species in the estuary. Letter a, b or c illustrates the significant differences between periods. The symbols refer to the decision tree (Fig 3) (TIF) [file pone.0173752.s003.tif]

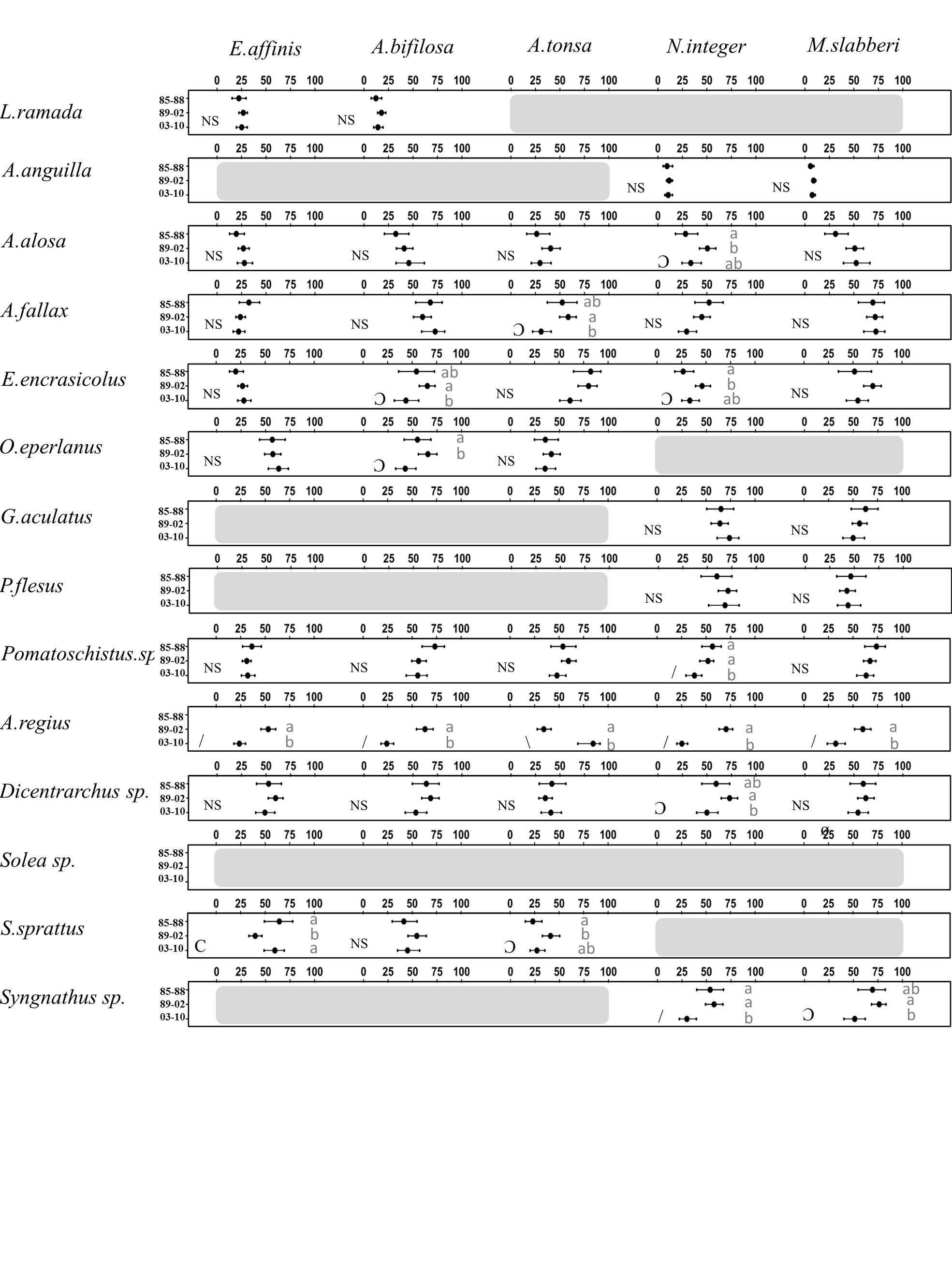

Supplement: S4 Fig — Intervals around the mean (black points) represent the intervals of credibility at 95%. Letter a, b or c illustrates the significant differences between periods. The symbols refer to the decision tree (Fig 3) (TIF) [file pone.0173752.s004.tif]
